# 

**Published:** 1875-09

**Authors:** 


### Books and Pamphlets Received.

Vision: Its Optical Defects and the Adaptation of Spectacles, with seventy-four illustrations on wood and selections from the test types of Jaeger and Snellen. By C. S. Fenner, M. D. Philadelphia: Lindsay & Blakiston, 1875. Buffalo: T. Butler & Son,

Transactions of the College of Physicians of Philadelphia. Third series, volume first. Philadelphia: Lindsay & Blakiston, 1875.

Transactions of the Medical Association of the State of Alabama, 1875.

Transactions of the Medical and Chirurgical Faculty of Maryland, 1875.

Uronology and its Practical Applications. By George M. Kober, M. D Reprint from the Richmond and Louisville *Medical Journal*, with illustrations.

Fracture of the Inferior Maxillary Bone. By Jos. F. Montgomery, M. D. Reprint from the Transactions of the California State Medical Society for 1875.

A Statement of the Relations of the Faculty of Medicine and Surgery of the University of Michigan to Homeopathy.

### Contents of September Number.

### ORIGINAL.

THE BUFFALO

### Medical and Surgical Journal

PUBLISHED MONTHLY,

Containing Original Articles, Reports of Medical Societies and Hospitals, Editorial, Reviews, Correspondence, Army News, etc., etc; including the usual variety of Medical Periodical Publications. Specimen copies sent on application.

Address Buffalo Medical & Surgical Journal, Buffalo, N. Y

TERMS OF SUBSCRIPTION, - $3.00 PER YEAR, IN ADVANCE.

